# Optic Disc Pit Maculopathy Treated With Human Amniotic Membrane and Autologous Internal Limiting Membrane Flap: A Case Report

**DOI:** 10.7759/cureus.102347

**Published:** 2026-01-26

**Authors:** Prasan Rao, Anupama Rao, Ansu Sara John, Jabeen Mohammed Jaffer

**Affiliations:** 1 Ophthalmology, Medcare Eye Centre, Dubai, ARE

**Keywords:** human amniotic membrane, internal limiting membrane flap, maculopathy, optic disc pit, retinal surgery, vitrectomy

## Abstract

Optic disc pit (ODP)-associated maculopathy represents a rare and challenging condition with variable response to treatment. We report a case of a 23-year-old woman who underwent pars plana vitrectomy (PPV) with placement of a human amniotic membrane (hAM) patch along with an autologous internal limiting membrane (ILM) flap to treat ODP-associated macular detachment. Initial postoperative outcomes demonstrated significant anatomical and functional improvement, which remained stable for one year. This report highlights the potential benefits of combined hAM-assisted repair with autologous ILM flap for optic disc pit maculopathy and underscores the need for long-term follow-up and further refinement of surgical techniques.

## Introduction

Optic disc pits (ODPs) are uncommon congenital excavations of the optic nerve head, classified within the spectrum of cavitary anomalies that also include optic disc coloboma, morning glory disc anomaly, and extrapapillary cavitations. The estimated prevalence of ODPs is approximately one in 11,000 individuals, with no significant sex predilection and a predominantly unilateral presentation [1]. In the early stages, affected patients may exhibit arcuate or step-shaped visual field defects, typically nasal or temporal, corresponding to localized loss of the retinal nerve fiber layer at the site of the pit. A subset of patients subsequently develops optic pit maculopathy (OPM), characterized by serous macular detachment and/or retinoschisis, which occurs in up to 50% of cases [1]. Various mechanisms have been proposed; however, none of them have been proven. Since OPM commonly occurs during the third and fourth decades of life, and progressive vitreous liquefaction starts approximately at the same time, vitreous traction may be related to OPM development. Another theory is that pressure gradient within the eye can lead to fluid migration from the vitreous cavity to the subretinal space. The origin of the fluid is also debatable. Fluid from the vitreous cavity, cerebrospinal fluid originating from the arachnoid space, fluid from leaky blood vessels at the base of the pit, and fluid from the orbital space surrounding the dura are considered as possible sources of the fluid. The visual prognosis of untreated OPM is generally poor, with most eyes progressing to a final visual acuity worse than 20/200, although occasional cases of spontaneous resolution have been reported [2]. To date, there is no standardized or universally accepted treatment strategy for OPM. Both nonsurgical and surgical interventions have been explored, yielding variable outcomes and a substantial rate of recurrence [3].

## Case presentation

**Figure 1:**
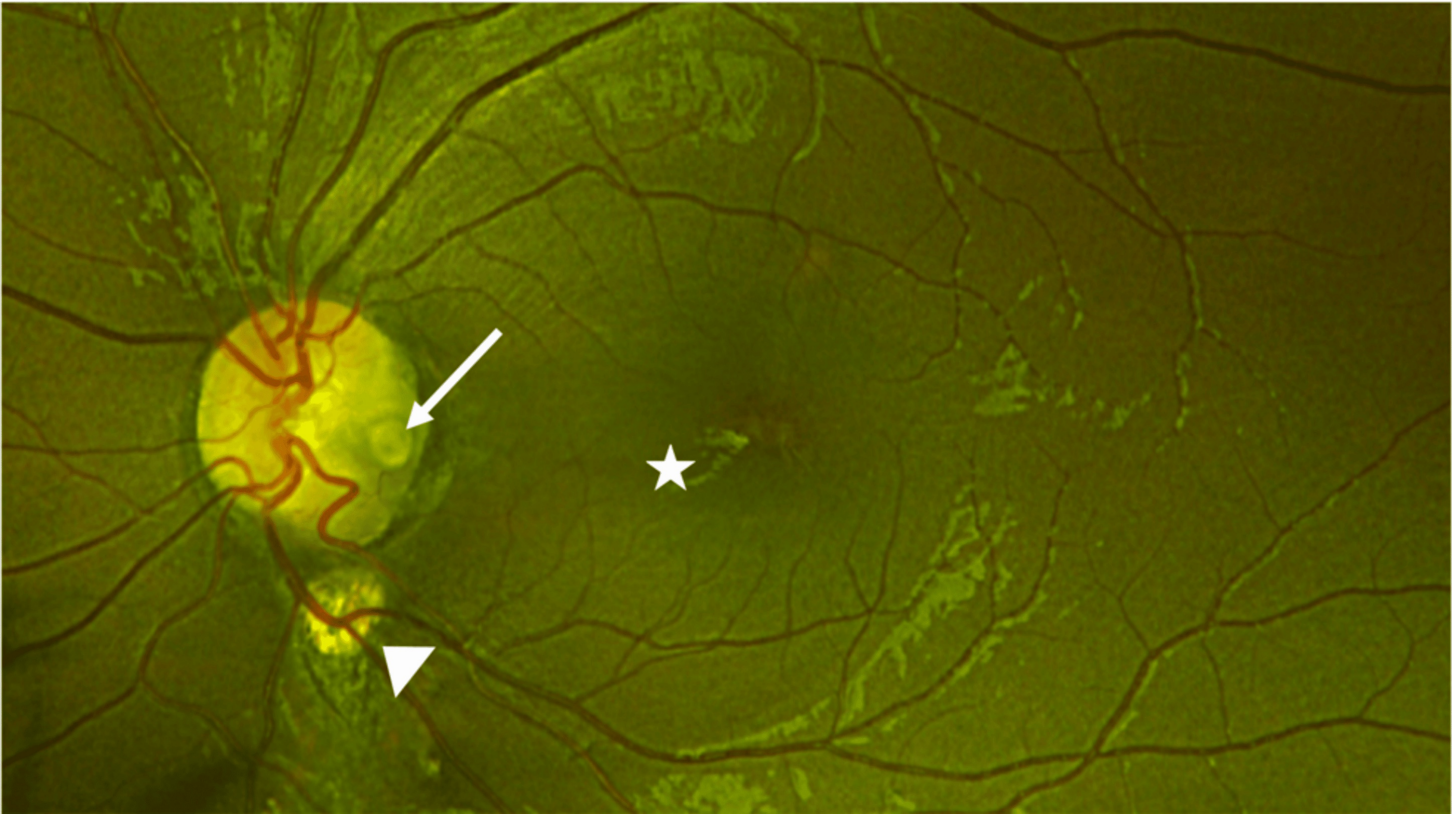
Pseudo-color fundus image of the left eye. This image shows the presence of an optic disc pit (white arrow) in the inferotemporal quadrant of the optic nerve head. Chorioretinal scar (white arrow head) is seen inferior to the disc. The left macula shows the presence of serous macular detachment (white star).

**Figure 2:**
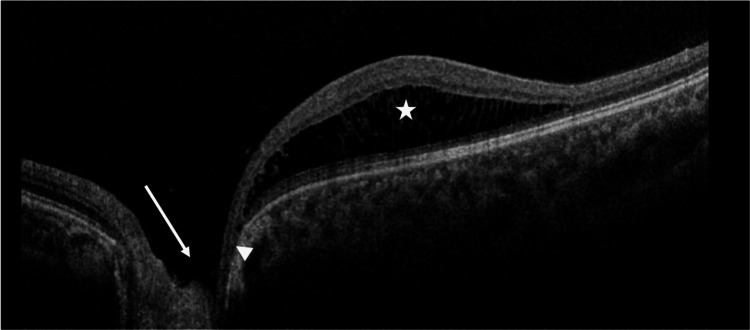
Spectral domain optical coherence tomography of the left eye. This OCT image shows the presence of an optic disc pit (white arrow). The communication (white arrow head) between the optic disc pit and schitic changes in the macula (white star) is visible in the scan. OCT: optical coherence tomography.

A 23-year-old woman with no systemic comorbidities presented with progressive visual loss in his left eye. Best-corrected visual acuity (BCVA) was 20/60p (Snellen vision) in the left eye and 20/20 (Snellen vision) in the fellow eye, which was structurally normal. Anterior segment examination was unremarkable. Fundus evaluation of the left eye revealed an optic disc pit in the inferotemporal quadrant of the optic nerve head, chorioretinal scar inferior to the disc, along with the serous macular detachment (Figure 1). The presence of a serous macular detachment with schitic changes communicating with the optic disc pit was confirmed on spectral-domain optical coherence tomography (OCT) (Figure 2). These macular changes were associated with increased central subfield thickness (CST) of 585 microns.

**Figure 3:**
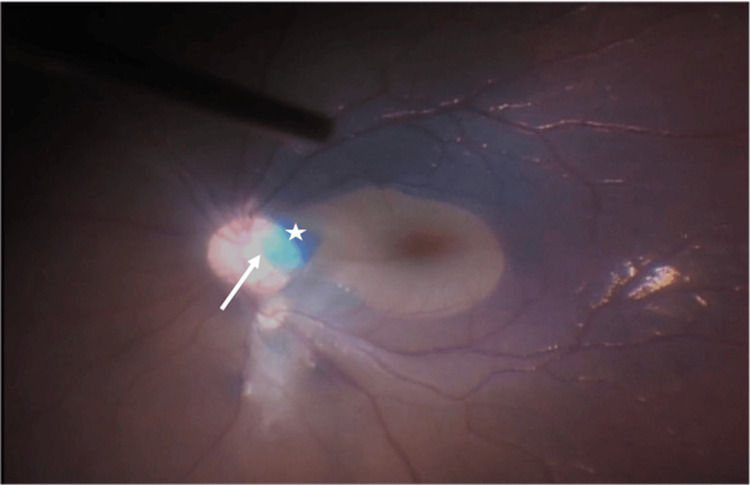
Intraoperative image of left optic disc. This image shows the presence of a human amniotic graft (hAM) (white arrow) with overlying autologous internal limiting membrane (ILM) flap (white star). The ILM flap was stained with ILM blue for better visualization.

Based on current recommendations [1,4], a 25-gauge pars plana vitrectomy was performed, with induction of the posterior vitreous detachment. A human amniotic membrane (hAM) patch graft was prepared by cutting a square-sized graft of approximately 1 mm × 1 mm size using a surgical scissor. Under chandelier illumination and using two intravitreal forceps, the hAM patch graft was positioned on the inferotemporal aspect of the optic disc to cover the optic disc pit, with the chorionic side towards the disc. The graft was gently stuffed to approximate the graft to the base of the optic disc pit. Two autologous internal limiting membrane (ILM) flaps were fashioned, with a hinge at the upper temporal and lower temporal edge of the optic disc. The hAM graft was stabilized with two autologous internal limiting membrane (ILM) flaps that were placed over it, without additional peripapillary laser photocoagulation. The surgery was completed with sulphur hexafluoride (SF6) gas tamponade (Figure 3).

**Figure 4:**
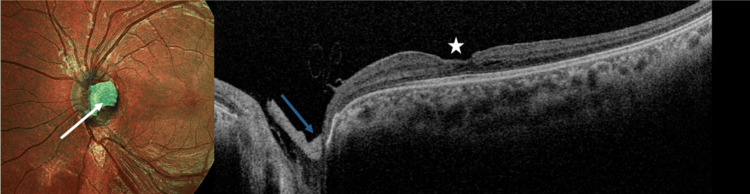
Postoperative pseudo-color image and OCT scan of the left eye. The pseudo-color image shows the hAM graft covering the optic disc (white arrow). OCT scan shows the hAM graft sealing the optic disc pit (blue arrow). Left macula shows resolution of the optic disc pit maculopathy with normalization of the foveal contour (white star). OCT: optical coherence tomography; hAM: human amniotic membrane.

The patient maintained a prone position for three days postoperatively. Follow-up examinations demonstrated progressive improvement in anatomy and function, with BCVA improving to 20/25p and 20/20p at four and eight months, respectively. OCT B-scan examination at four months revealed partial resolution of the schisis with CST of 412 microns. At the last follow-up examination (eight months), OCT B-scan examination showed a normal CST of 277 microns, with normalization of the foveal contour and complete resolution of macular schisis (Figure 4).

## Discussion

OPM is a complex and unpredictable entity in terms of pathophysiology and therapeutic response. The accumulation of subretinal and intraretinal fluid may arise from vitreous communication, cerebrospinal fluid migration, or leakage from peripapillary choroidal vessels [5-7]. While spontaneous resolution may rarely occur, most cases require surgical intervention [2]. Pars plana vitrectomy remains the mainstay of management, aimed at relieving vitreoretinal traction and reattaching the macula. Adjunctive procedures, such as ILM peeling, gas tamponade, or laser photocoagulation, are often performed to enhance anatomical success [3,8]. Recently, biological materials, including autologous ILM flaps, scleral plugs, and fibrin sealants, have been proposed to seal the pit, albeit with mixed durability [9].

Kohli et al. reported outcomes of 12 eyes with optic disc pit maculopathy (ODPM) managed using pars plana vitrectomy (PPV) employing a conventional surgical strategy that included induction of posterior vitreous detachment, selective internal limiting membrane (ILM) peeling, and C3F8 gas tamponade [10]. A statistically significant improvement in best-corrected visual acuity (BCVA) was observed, improving from 0.87 logMAR (Snellen equivalent 20/148) preoperatively to 0.38 logMAR (20/48) after surgery (p = 0.001), along with a corresponding reduction in central macular thickness (CMT). Analysis of optical coherence tomography (OCT) images demonstrated that eyes exhibiting outer retinal layer disruption experienced slower resolution of subretinal fluid (SRF) and achieved inferior visual outcomes. The authors postulated that postponement of surgical intervention may permit progressive outer retinal damage, thereby adversely affecting final visual recovery.

Formation of a postoperative macular hole has been described as a potential complication following pars plana vitrectomy (PPV) with internal limiting membrane (ILM) peeling in eyes with optic disc pit maculopathy (ODPM). To mitigate this risk, several surgeons have adopted fovea-sparing surgical strategies. In this context, D’Souza et al. reported outcomes in 10 patients with ODPM who underwent PPV combined with a fovea-sparing inverted ILM flap placed toward the optic disc, followed by gas tamponade using either SF6 or C3F8 [11]. At 12 months of follow-up, mean best-corrected visual acuity (BCVA) improved significantly from 0.91 logMAR (Snellen equivalent 20/162) preoperatively to 0.58 logMAR (20/76) (p = 0.008), accompanied by a significant reduction in central macular thickness (CMT). Anatomical resolution of maculopathy was complete in 70% of cases, while the remaining 30% exhibited persistent shallow subretinal fluid (SRF). Notably, none of the treated eyes developed a postoperative macular hole.

Muftuoglu et al. described outcomes from a small case series of patients with optic disc pit maculopathy (ODPM) treated using pars plana vitrectomy (PPV) combined with conventional internal limiting membrane (ILM) plugging and C3F8 gas tamponade [12]. The surgical technique involved induction of posterior vitreous detachment, followed by ILM peeling and insertion of the peeled membrane into the optic disc pit, with perfluorocarbon liquid used to assist flap positioning. The authors emphasized a potential risk associated with perfluorocarbon use in eyes with optic disc pits, as the heavy liquid may inadvertently track through the pit into the subarachnoid space. Following surgery, complete resolution of intra- and subretinal fluid was achieved in four of six eyes within five months. Visual outcomes showed a statistically significant improvement, with best-corrected visual acuity (BCVA) improving from 1.25 to 0.86 logMAR (Snellen equivalent 20/355 to 20/144; p = 0.043).

Caporossi et al. described outcomes in 11 eyes with optic disc pit maculopathy (ODPM), including nine treatment-naïve cases and two eyes with persistent disease following prior pars plana vitrectomy (PPV), managed using PPV combined with human amniotic membrane (hAM) grafting [13]. The surgical protocol involved PPV with induction of posterior vitreous detachment, followed by placement of a 1.0-1.5 mm hAM patch within the optic disc pit. The graft was secured intraoperatively using perfluorocarbon liquid during fluid-air exchange, after which air tamponade was performed. Patients were advised to maintain prone positioning for three days postoperatively. At one-year follow-up, mean best-corrected visual acuity (BCVA) improved significantly from 0.58 logMAR (Snellen equivalent 20/76) to 0.16 logMAR (20/29) (p < 0.00001). Anatomically, complete resolution of intra- and subretinal fluid was achieved in 81.8% of eyes, while the remaining 18% demonstrated partial fluid reduction without recurrence. No intraoperative or postoperative complications were encountered, including hAM displacement or retinal detachment.

The application of human amniotic membrane (hAM) offers an innovative and biocompatible solution due to its anti-inflammatory and neurotrophic properties [14-16]. However, late recurrences have been described by Vaiano et al., emphasizing the need for extended follow-up and technique refinement. The combination of hAM along with an autologous ILM flap offers long-term graft integration. In this case, the visual outcome in the long-term was favorable, with no recurrences [17]. 

## Conclusions

Human amniotic membrane-assisted repair combined with autologous ILM flaps following pars plana vitrectomy appears to be a promising therapeutic approach for ODP-related maculopathy, demonstrating favorable short-term anatomical and visual results. This technique may offer durability of the biological sealing method, but further studies to optimize the surgical technique are necessary.
